# Efficacy of Atezolizumab Plus Bevacizumab–Transcatheter Arterial Chemoembolization Sequential Therapy for Patients with Intermediate-Stage Hepatocellular Carcinoma

**DOI:** 10.3390/curroncol31100432

**Published:** 2024-09-27

**Authors:** Etsuko Moriyama, Shigeo Shimose, Takashi Niizeki, Hideki Iwamoto, Masatoshi Tanaka, Tomotake Shirono, Yu Noda, Masahito Nakano, Ryoko Kuromatsu, Hironori Koga, Takumi Kawaguchi

**Affiliations:** 1 Division of Gastroenterology, Department of Medicine, Kurume University School of Medicine, Fukuoka 830-0011, Japan; moriyama_etsuko@kurume-u.ac.jp (E.M.); niizeki_takashi@kurume-u.ac.jp (T.N.); iwamoto_hideki@med.kurume-u.ac.jp (H.I.); shirono_tomotake@med.kurume-u.ac.jp (T.S.); noda_yuu@med.kurume-u.ac.jp (Y.N.); nakano_masahito@kurume-u.ac.jp (M.N.); ryoko@med.kurme-u.ac.jp (R.K.); hirokoga@med.kurume-u.ac.jp (H.K.); takumi@med.kurume-u.ac.jp (T.K.); 2 Iwamoto Internal Medical Clinic, Kitakyusyu 802-0832, Japan; 3 Clinical Research Center, Yokokura Hospital, Miyama, Fukuoka 839-0295, Japan; mazzo6528@me.com

**Keywords:** atezolizumab plus bevacizumab, hepatocellular carcinoma, TACE

## Abstract

This retrospective study aimed to evaluate the impact of atezolizumab plus bevacizumab–transcatheter arterial chemoembolization (TACE) sequential therapy in unresectable hepatocellular carcinoma (HCC), especially in patients with intermediate-stage HCC. A total of 212 patients were enrolled and categorized into the Atez/Bev-TACE sequential therapy (*n* = 23) or Atez/Bev monotherapy group (*n* = 189) between 2020 and 2024. Of these, patients with intermediate-stage HCC were categorized into the Atez/Bev-TACE sequential (*n* = 18) or Atez/Bev monotherapy group (*n* = 91). The best objective response rate, disease control rate, and median progression-free survival (PFS) after TACE were 73.9%, 82.6%, and 6.1 months, respectively. The PFS after TACE was significantly higher in the Atez/Bev sequential therapy group than in the no-Atez/Bev-administration group after TACE (6.9 months vs. 5.0 months, *p* = 0.025). The median overall survival (OS) was significantly higher in the Atez/Bev-TACE sequential therapy group than in the Atez/Bev monotherapy group for intermediate-stage HCC (34.9 months vs. 17.8 months; *p* = 0.016). Independent factors associated with OS were low alpha-fetoprotein levels, modified albumin–bilirubin 1 or 2a levels, and Atez/Bev-TACE sequential therapy. Atez/Bev-TACE sequential therapy improved prognosis compared with Atez/Bev monotherapy in patients with intermediate-stage HCC. Moreover, Atez/Bev should be readministered after TACE.

## 1. Introduction

Hepatocellular carcinoma (HCC) is one of the most prevalent malignancies globally and is a leading cause of cancer-related death [1]. Over half of patients diagnosed at intermediate-to-advanced stages have unresectable HCC (u-HCC), hence missing the opportunity for curative therapy [2,3]. These patients are only suitable for palliative treatments such as transcatheter arterial chemoembolization (TACE) and systemic therapy [4]. Systemic therapy with molecular-targeted agents, such as sorafenib [5] and lenvatinib (LEN) [6], has been remarkably developed in the last few years [7]. Immune checkpoint inhibitors have recently revolutionized u-HCC treatment strategies. However, the prognosis of patients with u-HCC remains unsatisfactory, and improved therapeutic strategies for systemic therapy are required to overcome this poor prognosis.

The IMbrave150 trial, a global phase 3 clinical trial, showed that combination therapy of atezolizumab plus bevacizumab (Atez/Bev), in which monoclonal antibodies bind to programmed cell death 1-ligand 1 (PD-L1) and antivascular endothelial growth factor (VEGF)-A, is the recommended first-line treatment for patients with u-HCC [8]. The results of the updated IMbrave150 trial also showed that Atez/Bev treatment revealed that the progression-free survival (PFS) and overall survival (OS) were significantly improved compared with sorafenib in patients with u-HCC [9].

According to multiple guidelines, TACE is the recommended standard treatment for patients with intermediate-stage HCC according to multiple guidelines [10,11]. However, the effectiveness of TACE alone is often limited because of the high prevalence of HCC recurrence and the high heterogeneity of intermediate-stage liver cancer [12]. Thus, systemic therapy has been recommended for some patients with intermediate-stage HCC [13,14]. Recently, some studies have shown that combining TACE with other systemic therapies can improve the prognosis of patients with u-HCC [15,16] and that systemic therapy combined with TACE has been the dominant strategy for intermediate-stage HCC. TACE increases tumor hypoxia, leading to the upregulation of VEGF expression and induced tumor angiogenesis [17]. In addition, TACE increases the release of tumor neoantigens, leading to an increased expression of PD-L1 [18]. Thus, Atez/Bev treatment combined with TACE may be promising for patients with intermediate-stage HCC. However, there have been few reports on the efficacy of Atez/Bev-TACE sequential therapy, and the associated outcomes remain unclear.

This study aimed to investigate the efficacy of Atez/Bev sequential therapy in patients with u-HCC, especially with intermediate-stage HCC.

## 2. Materials and Methods

### 2.1. Study Design and Patients

**Figure 1:**
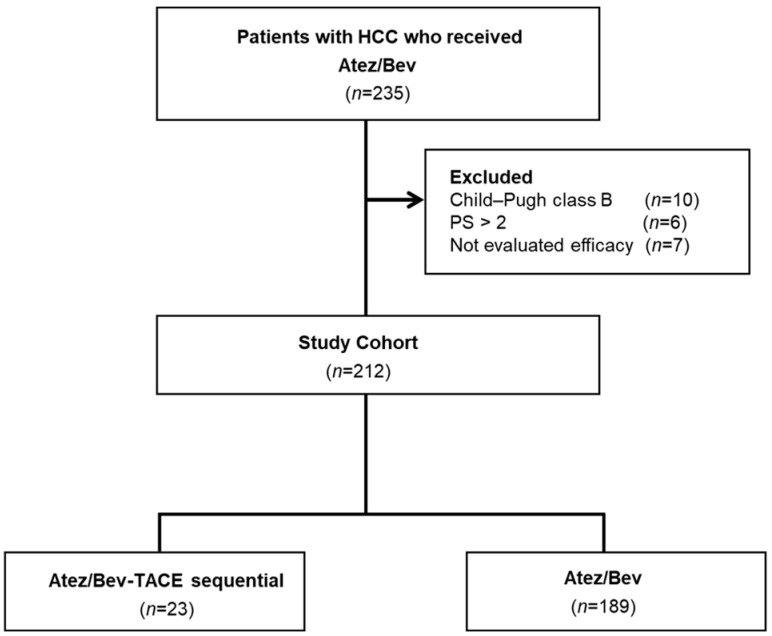
Study flowchart.

The data of 235 patients treated with Atez/Bev for u-HCC between November 2020 and June 2024 were retrospectively evaluated. The exclusion criteria for this study were as follows: *n* = 23; Child–Pugh class B, 10 patients; Eastern Cooperative Oncology Group performance status <2, 6 patients; not evaluated for efficacy, 7 patients. A total of 212 patients were enrolled and classified into the Atez/Bev-TACE sequential therapy group (n = 23) or the Atez/Bev monotherapy group (*n* = 189) (Figure 1). This study was approved by the Institutional Review Board of Kurume University School of Medicine (approval code: 20183).

### 2.2. Assessment of Hepatic Reserve Function

Liver function was evaluated using the Child–Pugh classification [19] and albumin–bilirubin (ALBI) scores [20]. The ALBI score was calculated based on serum albumin and total bilirubin levels. Based on the ALBI score, liver function was assessed using the modified ALBI (mALBI) grade. ALBI grade 2 is divided into 2a and 2b, with an ALBI score of −2.27 as the cutoff value [20].

### 2.3. Atez/Bev Treatment Protocol

According to pharmaceutical recommendations, patients were intravenously administered atezolizumab (1200 mg) and bevacizumab (7.5 mg/kg) every three weeks. Treatment persisted until tumor progression or the emergence of insufferable adverse events (AEs). AEs were graded using the National Cancer Institute Common Terminology Criteria for Adverse Events version 5.0 [21]. Atezolizumab was discontinued when unacceptable immune-related AEs occurred, and these patients received bevacizumab monotherapy. In turn, bevacizumab was discontinued when unacceptable bevacizumab-related AEs occurred, and these patients received atezolizumab monotherapy.

### 2.4. Evaluation of Therapeutic Efficacy of Atez/Bev Treatment

To evaluate the therapeutic response, the Modified Response Evaluation Criteria in Solid Tumors (mRECIST) criteria [22] was employed using dynamic computed tomography or magnetic resonance imaging at approximately 6 weeks after the initiation of treatment with Atez/Bev. Thereafter, it was performed every 3 weeks until death or study cessation.

### 2.5. Atez/Bev-TACE Sequential Therapy

In cases where a therapeutic response to Atez/Bev treatment was detected, treatment was continued. However, for cases where tumor vascularity resumed after having disappeared previously; where the tumor progressed after having once shrunk, such as nodules, for which Atez/Bev has limited effect [23]; or in cases in which alpha-fetoprotein (AFP) levels increased despite no progression on imaging evaluation, we administered the recommended Atez/Bev-TACE sequential therapy to patients with tolerance to Atez/Bev and TACE, considering these cases comprehensively. TACE was conducted 3 weeks after the completion of Atez/Bev treatment and was restarted approximately 3 weeks after TACE depending on the patient’s condition. In contrast, patients with conditions in which TACE is expected to achieve cancer-free status did not receive subsequent Atez/Bev therapy due to the drug-free purpose, with informed consent provided.

### 2.6. TACE Treatment Protocol

The chemoembolization procedure followed the technical details described previously [24]. A microcatheter was introduced into the tumor-feeding artery. TACE was performed using epirubicin or cisplatin with lipiodol depending on the tumor size and injection of gelatin sponge particles to embolize the tumor-feeding vessels.

### 2.7. Statistical Analysis

Continuous variables were compared using a one-way analysis of variance with Scheffe’s post hoc test, and categorical variables were compared between groups using the χ2 test or Fisher’s exact analysis. The PFS and OS were calculated using the Kaplan–Meier method and compared by the log-rank test. A swimmer plot was used to describe the history of the investigator’s assessment. Factors associated with the OS were also analyzed using a Cox proportional hazard regression analysis model. *p* values of less than 0.05 were considered as denoting statistical significance. All statistical analyses were performed using JMP Pro version 15 (SAS Institute Inc., Cary, NC, USA).

## 3. Results

### 3.1. Patient Characteristics

Table 1 summarizes the baseline characteristics of the study participants. The patients’ median age was 73 years, and 21.2% (45/212) of the patients were female. Non-viral HCC was observed in 86 patients (40.5%). The median ALBI score was −2.44, and the mALBI grade was 1 in 71 patients (33.5%). Macrovascular invasion and extrahepatic spread were observed in 39 (18.3%) and 72 (33.9%) patients, respectively. Among these patients, 143 (67.5%) and 69 (32.5%) received first- and later-line treatments, respectively. Atez/Bev-TACE sequential therapy was performed in 10.8% (23/212) of patients, with an average of seven Atez/Bev cycles before TACE. Barcelona Clinic Liver Cancer stage and extrahepatic spread were significantly different in the Atez/Bev monotherapy group compared to the Atez/Bev-TACE sequential therapy group; there were no other significant differences between groups.

### 3.2. Swimmer Plot Analysis Assessment by Investigator Assessment

**Figure 2:**
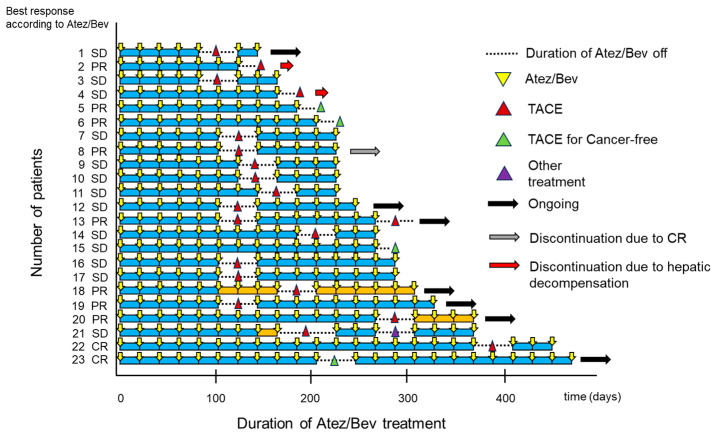
Swimmer plot of investigator assessment in the Atez/Bev-TACE sequential therapy group. Yellow triangles represent an Atez/Bev treatment cycle. Reddish-brown triangles represent TACE. Dark blue arrows represent ongoing treatment with Atez/Bev-TACE. Green triangles represent added TACE for achieving cancer-free status.

The swimmer plot for patients treated with Atez/Bev-TACE sequential therapy is shown in Figure 2. Twenty-three patients were treated with Atez/Bev-TACE sequential therapy, and six patients continued treatment based on the cutoff data.

### 3.3. Best Therapeutic Response of Atez/Bev-TACE Sequential Therapy According to mRECIST Criteria

The distribution of therapeutic responses to Atez/Bev-TACE is shown in Table A1. Complete response, partial response (PR), stable disease (SD), and progressive disease were observed in 6 (26.1%), 11 (47.8%), 2 (8.7%), and 4 patients (17.3%), respectively. The overall objective response rate (ORR) and disease control rate (DCR) were 73.9% and 82.6%, respectively.

### 3.4. PFS after TACE and Comparison of PFS between Patients with and without Atez/Bev Administration after TACE

**Figure 3:**
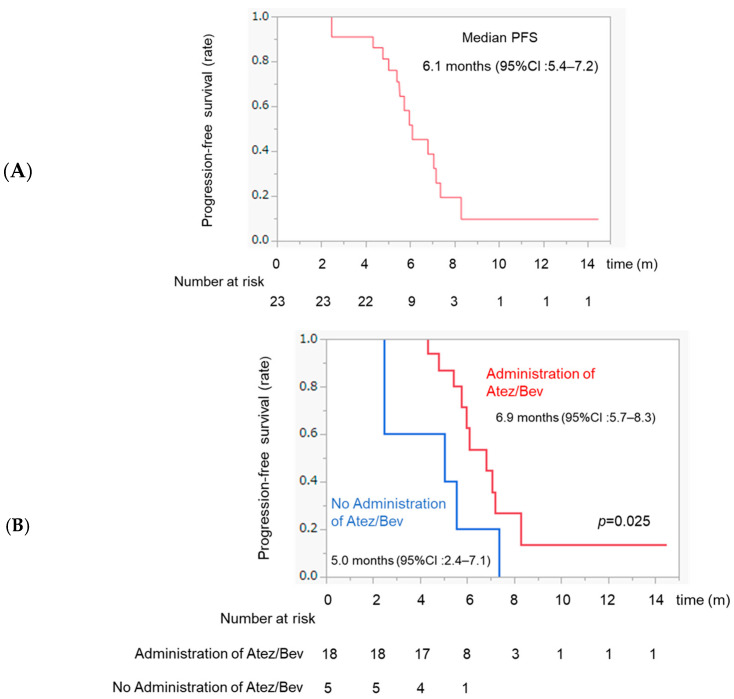
Kaplan–Meier curves for progression-free survival. (**A**) Progression-free survival from TACE. (**B**) Progression-free survival according to the administration of Atez/Bev after TACE. The red line indicates the administration of Atez/Bev after TACE. The blue line indicates no administration of Atez/Bev after TACE.

The median PFS was 6.1 months after TACE according to the mRECIST criteria (Figure 3A). The PFS was significantly higher in the Atez/Bev administration after TACE group than that in the no-Atez/Bev-administration group (6.9 months vs. 5.0 months, *p* = 0.025) (Figure 3B).

### 3.5. Changes in the ALBI Score in the Atez/Bev-TACE Sequential Treatment Group

**Figure A1:**
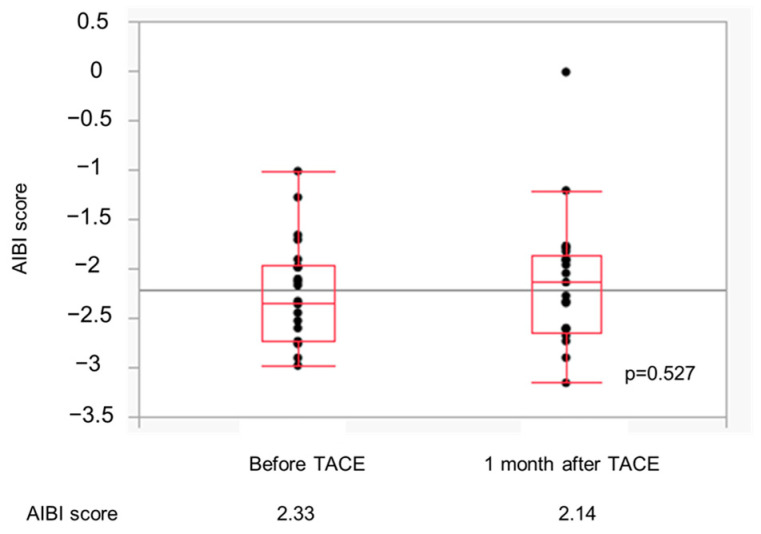
Change in ALBI score from baseline to 4 weeks after TACE.

Figure A1 shows the changes in ALBI scores from baseline to 4 weeks after TACE. The median ALBI scores at baseline and 4 weeks after TACE were −2.33 and −2.14, respectively. There was no deterioration in the ALBI scores (−2.33 vs. −2.14, *p* = 0.527).

### 3.6. Patient Characteristics in Patients with Intermediate-Stage HCC

Table 2 summarizes the characteristics of patients with intermediate-stage HCC. In total, 109 patients were included in this study. The patients’ median age was 74 years, and 22.0% of patients were female. The within up-to-seven criteria characteristic was observed in 21.1% (23/109) of patients. The median ALBI score was −2.46, and the mALBI grade was 1 in 38 patients (34.8%). The average number of Atez/Bev cycles administered prior to TACE was 10. There were no significant differences between the Atez/Bev-TACE sequential therapy and monotherapy groups.

### 3.7. Comparison of OS between Atez/Bev and Atez/Bev-TACE Sequential Therapy in the Whole Sample and in Patients with Intermediate-Stage HCC

**Figure 4:**
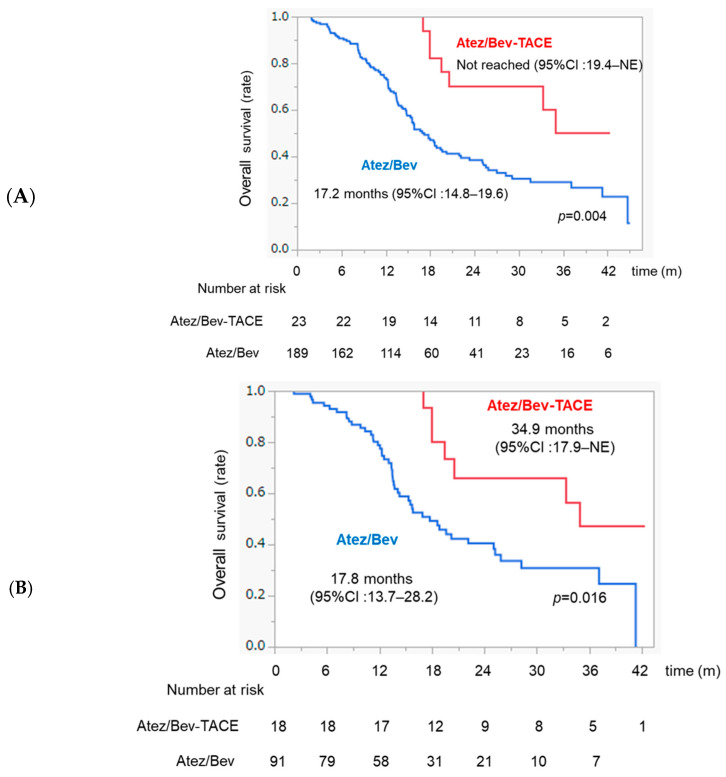
Overall survival time in patients with HCC treated with Atez/Bev. (**A**) Kaplan–Meier curves for overall survival in the Atez/Bev-TACE sequential therapy group and the Atez/Bev monotherapy group. (**B**) Kaplan–Meier curves for overall survival in the Atez/Bev-TACE sequential therapy group and the Atez/Bev monotherapy group in patients with intermediate-stage HCC.

In the whole sample, the OS was significantly higher in the Atez/Bev-TACE sequential therapy group than in the Atez/Bev monotherapy group (not reached vs. 17.2 months, *p* = 0.004) (Figure 4A). In patients with intermediate-stage HCC, the OS was significantly higher in the Atez/Bev-TACE sequential therapy group than in the Atez/Bev monotherapy group (34.9 months vs. 17.8 months, *p* = 0.016, respectively) (Figure 4B).

### 3.8. Univariate and Multivariate Analyses of Factors Associated with OS in Patients with Intermediate-Stage HCC

mALBI grade 1 or 2a, tumor size < 30 mm, AFP level < 400 ng/mL, and Atez/Bev TACE sequential therapy were selected as variables via univariate analysis. In the multivariate analysis, mALBI grade 1 or 2a, AFP < 400, and Atez/Bev-TACE sequential therapy were identified as independent factors associated with OS (Table 3).

## 4. Discussion

This study indicated that Atez/Bev-TACE sequential therapy significantly prolonged survival compared to Atez/Bev monotherapy in patients with intermediate-stage HCC. Moreover, although Atez/Bev-TACE sequential therapy was identified as an independent predictive factor for OS, Atez/Bev should be readministered after TACE.

The present study found an ORR and DCR of 73.9% and 82.6%, respectively, in patients with intermediate-stage HCC after TACE. A previous study reported that ORR and DCR were 66.7% and 92.9% after TACE combined with Atez/Bev [25], which is consistent with our study. The most important timing of additional TACE is tumor progression after having shrunk once or cases in which the AFP value is elevated despite no progression on imaging evaluation. Controlling the intrahepatic tumor burden is particularly important as it may preserve liver function, extend the duration of systemic therapy, and improve the prognosis of patients with u-HCC [26,27]. Moreover, we previously reported the inconsistency between radiological findings and changes in the AFP levels of PR and SD cases in patients undergoing Atez/Bev treatment [28]. Kudo et al. also reported that within the SD response group, the shrinkage group had a significantly longer OS than the no-shrinkage group [29]. In brief, when the AFP level is elevated despite no progression on imaging evaluation, such as SD or PR, the addition of TACE may be a promising combination treatment strategy.

In this study, we found that the PFS was significantly higher in the group with Atez/Bev readministration after TACE than in the group with no Atez/Bev administration after TACE. In this study, all three patients who discontinued Atez/Bev despite undergoing TACE to achieve a cancer-free status had early tumor progression (Figure 2). The disadvantages of TACE include inducing the upregulation of HIF1-α and upregulating the expression of VEGF, fibroblast growth factor, or platelet-derived growth factor, followed by increasing tumor angiogenesis [30]. These factors may be associated with residual survival of neovascularization-rich HCC tissue after TACE treatment. On the positive side, TACE is expected to attack cancer cells and boost immune activation and recognition [31]. Therefore, it is reasonable to readminister Atez/Bev after TACE. More importantly, TACE must not be performed on all nodules but only on a selection of target nodules to preserve liver function [23]. This is because performing TACE on all nodules may cause deterioration of liver function, making it impossible to readminister Atez/Bev. In fact, Atez/Bev could not be readministered in two patients due to deterioration of liver function after TACE (Figure 2). Therefore, TACE should be performed under the assumption that Atez/Bev will be readministered.

Our findings demonstrated that low AFP levels, mALBI grade 1 or 2a, and Atez/Bev-TACE sequential therapy were independent prognostic factors for patients with intermediate-stage HCC treated with Atez/Bev. It is well known that elevated AFP values are associated with poor prognosis across all stages of HCC [13], and preserved liver function is associated with OS in Atez/Bev treatment [32]. Notably, Atez/Bev-TACE sequential therapy significantly prolonged the prognosis of patients with intermediate-stage HCC compared to Atez/Bev monotherapy in this study. Recently, the efficacy of the combination of TACE with immune checkpoint inhibitors or molecular targeting agents has been reported [16,33]. The EMERALD-1 trial showed that the combination treatment of TACE with durvalumab and bevacizumab prolonged PFS compared with TACE alone [34], and the LEAP-012 study showed that a combination of lenvatinib plus pembrolizumab plus TACE therapy significantly improved PFS compared to placebo plus TACE in patients with intermediate-stage HCC [35]. Moreover, the IMPACT trial, a randomized controlled trial comparing Atez/Bev plus on-demand TACE to Atez/Bev alone in patients with an SD response, is ongoing in Japan [36]. In the future, a combination of TACE and systemic therapy may become the standard treatment strategy for patients with intermediate-stage HCC.

The limitations of this study were as follows. First, the study design was conducted retrospectively. Second, the amount of Atez/Bev-TACE sequential therapy was small. Third, selection bias existed in the classification of the Atez/Bev-TACE sequential therapy and Atez/Bev monotherapy groups. Therefore, a randomized controlled prospective validation study is required with a large number of patients with intermediate-stage HCC to determine the efficacy of Atez/Bev-TACE sequential therapy.

## 5. Conclusions

In conclusion, we demonstrated that Atez/Bev-TACE sequential therapy improved prognosis compared to Atez/Bev monotherapy in patients with intermediate-stage HCC. Moreover, Atez/Bev should be readministered after TACE.

## Figures and Tables

**Table 1:** Patient characteristics.

Characteristic	All Patients	Atez/ Bev-TACE	Atez/Bev	*p*
n	212	23	189	
Age (years old)	73 (37–93)	69 (50–84)	73 (37–93)	0.255
Sex (female/male)	45/167	5/18	40/149	0.893
PS (0/1/)	182/30	19/4	163/26	0.636
Body Mass Index (kg/m^2^)	23.0 (15.9–35.3)	23.9 (15.4–35.3)	23.1 (15.9–35.2)	0.091
Etiology (viral/non-viral)	126/86	12/11	114/75	0.452
ALBI score (Median (range))	−2.44 (−3.50–−1.55)	−2.45 (−2.96–−1.71)	−2.44 (−3.50–−1.55)	0.964
mALBI grade (1/2a/2b)	71/76/65	10/6/7	61/70/58	0.483
BCLC stage (A/B/C)	2/109/101	1/18/4	1/91/97	0.003
tumor size (mm)	32 (10–136)	26 (11–132)	32.0 (10–136)	0.255
Number of tumors <5/≥5	74/138	9/14	65/124	0.652
Macrovascular invasion (No/Yes)	173/39	21/2	152/37	0.203
Extrahepatic spread (No/Yes)	140/72	20/3	120/69	0.016
AFP (ng/mL)	32.1 (1.2–284, 543)	6.4 (1.4–1167)	36.5 (1.2–209, 295)	0.082
Number of Atez/Bev cycles before TACE		7 (5–19)		n.s
Treatment line (first-line/later-lines)	143/69	13/10	130/59	0.236

**Table 2:** Patient characteristics in patients with intermediate-stage HCC.

Characteristic	All Patients	Atez/Bev-TACE	Atez/Bev	*p*
n	109	18	91	
Age (years old)	74 (50–93)	71 (50–84)	75 (51–93)	0.219
Sex (female/male)	24/85	5/13	19/72	0.518
Body Mass Index (kg/m^2^)	23.0 (16.6–35.3)	23.2 (19.5–29.6)	23.0 (16.6–35.2)	0.641
Etiology (viral/non-viral)	62/47	11/7	51/40	0.691
ALBI score (Median (range))	−2.46 (−3.50–−1.59)	−2.47 (−2.92–−1.70)	−2.44 (−3.50–−1.59)	0.873
mALBI grade (1/2a/2b)	38/35/36	8/4/6	30/31/30	0.542
Up-to-seven (in/out)	23/86	4/14	19/72	0.895
tumor size (mm)	32 (10–136)	25.0 (11–132)	30.0 (10–136)	0.285
Number of tumors <5/≥5	36/73	7/11	29/62	0.562
AFP (ng/mL)	11.4 (1.2–129, 743)	10.7 (1.4–1167)	11.4 (1.2–129, 743)	0.725
Number of Atez/Bev cycles before TACE		10 (6–19)		
Treatment line (first-line/later-lines)	74/35	11/7	63/28	0.500

**Table 3:** Univariate and multivariate analyses of factors associated with OS.

Variable	Univariate Analysis	Multivariate Analysis		
	*p*-Value	Odds Ratio	95% CI	*p*-Value
Age, <70 vs. ≥70	0.615			
Sex, female vs. male	0.800			
Etiology, viral vs. non-viral	0.355			
mALBI grade, 1 or 2a vs. 2b	<0.001	0.311	0.175–0.553	<0.001
Up-to-seven (in/out)	0.048	0.729	0.353–1.502	0.391
Number of tumors, <5 vs. ≥5	0.881			
Tumor size, <30 vs. ≥30	0.017	0.696	0.385–1.256	0.228
AFP, <400 vs. ≥400 ng/mL	0.001	0.228	0.116–0.449	<0.001
Treatment line, (first-line vs. later-lines)	0.094			
Atez/Bev-TACE sequential, (+/−)	0.015	0.311	0.138–0.702	0.001

## Data Availability

Data that support the findings of this study are available from the author, S.S. (Shigeo Shimose), on reasonable request.

## Appendix A

**Table A1:** Best response rate according to modified-RECIST after TACE.

Response Category	m-RECIST
CR	6 (26.1%)
PR	11 (47.8%)
SD	2 (8.7%)
PD	4 (17.3%)
ORR	17 (73.9%)
DCR	19 (82.6%)

## References

[1] Rumgay H., Arnold M., Ferlay J., Lesi O., Cabasag C.J., Vignat J., Laversanne M., McGlynn K.A., Soerjomataram I. (2022). Global Burden of Primary Liver Cancer in 2020 and Predictions to 2040. J. Hepatol..

[2] Fu Z., Li X., Zhong J., Chen X., Cao K., Ding N., Liu L., Zhang X., Zhai J., Qu Z. (2021). Lenvatinib in Combination with Transarterial Chemoembolization for Treatment of Unresectable Hepatocellular Carcinoma (Uhcc): A Retrospective Controlled Study. Hepatol. Int..

[3] Villanueva A. (2019). Hepatocellular Carcinoma. N. Engl. J. Med..

[4] Llovet J.M., Villanueva A., Marrero J.A., Schwartz M., Meyer T., Galle P.R., Lencioni R., Greten T.F., Kudo M., Mandrekar S.J. (2021). Trial Design and Endpoints in Hepatocellular Carcinoma: Aasld Consensus Conference. Hepatology.

[5] Llovet J.M., Ricci S., Mazzaferro V., Hilgard P., Gane E., Blanc J.F., de Oliveira A.C., Santoro A., Raoul J.L., Forner A. (2008). Sorafenib in Advanced Hepatocellular Carcinoma. N. Engl. J. Med..

[6] Kudo M., Finn R.S., Qin S., Han K.H., Ikeda K., Piscaglia F., Baron A., Park J.W., Han G., Jassem J. (2018). Lenvatinib Versus Sorafenib in First-Line Treatment of Patients with Unresectable Hepatocellular Carcinoma: A Randomised Phase 3 Non-Inferiority Trial. Lancet.

[7] Kudo M. (2020). Management of Hepatocellular Carcinoma in Japan: Current Trends. Liver Cancer.

[8] Finn R.S., Qin S., Ikeda M., Galle P.R., Ducreux M., Kim T.Y., Kudo M., Breder V., Merle P., Kaseb A.O. (2020). Atezolizumab Plus Bevacizumab in Unresectable Hepatocellular Carcinoma. N. Engl. J. Med..

[9] Cheng A.L., Qin S., Ikeda M., Galle P.R., Ducreux M., Kim T.Y., Lim H.Y., Kudo M., Breder V., Merle P. (2022). Updated Efficacy and Safety Data from Imbrave150: Atezolizumab Plus Bevacizumab Vs. Sorafenib for Unresectable Hepatocellular Carcinoma. J. Hepatol..

[10] Forner A., Reig M., Bruix J. (2018). Hepatocellular Carcinoma. Lancet.

[11] Omata M., Cheng A.L., Kokudo N., Kudo M., Lee J.M., Jia J., Tateishi R., Han K.H., Chawla Y.K., Shiina S. (2017). Asia-Pacific Clinical Practice Guidelines on the Management of Hepatocellular Carcinoma: A 2017 Update. Hepatol. Int..

[12] Bolondi L., Burroughs A., Dufour J.F., Galle P.R., Mazzaferro V., Piscaglia F., Raoul J.L., Sangro B. (2012). Heterogeneity of Patients with Intermediate (Bclc B) Hepatocellular Carcinoma: Proposal for a Subclassification to Facilitate Treatment Decisions. Semin. Liver Dis..

[13] Reig M., Forner A., Rimola J., Ferrer-Fàbrega J., Burrel M., Garcia-Criado Á., Kelley R.K., Galle P.R., Mazzaferro V., Salem R. (2022). Bclc Strategy for Prognosis Prediction and Treatment Recommendation: The 2022 Update. J. Hepatol..

[14] Shimose S., Iwamoto H., Tanaka M., Niizeki T., Shirono T., Noda Y., Kamachi N., Okamura S., Nakano M., Suga H. (2021). Alternating Lenvatinib and Trans-Arterial Therapy Prolongs Overall Survival in Patients with Inter-Mediate Stage Hepatocellular Carcinoma: A Propensity Score Matching Study. Cancers.

[15] Kudo M., Ueshima K., Ikeda M., Torimura T., Tanabe N., Aikata H., Izumi N., Yamasaki T., Nojiri S., Hino K. (2020). Randomised, Multicentre Prospective Trial of Transarterial Chemoembolisation (Tace) Plus Sorafenib as Compared with Tace Alone in Patients with Hepatocellular Carcinoma: Tactics Trial. Gut.

[16] Kudo M., Ueshima K., Saeki I., Ishikawa T., Inaba Y., Morimoto N., Aikata H., Tanabe N., Wada Y., Kondo Y. (2024). A Phase 2, Prospective, Multicenter, Single-Arm Trial of Transarterial Chemoembolization Therapy in Combination Strategy with Lenvatinib in Patients with Unresectable Intermediate-Stage Hepatocellular Carcinoma: Tactics-L Trial. Liver Cancer.

[17] Carmeliet P., Jain R.K. (2000). Angiogenesis in Cancer and Other Diseases. Nature.

[18] Tischfield D.J., Gurevich A., Johnson O., Gatmaytan I., Nadolski G.J., Soulen M.C., Kaplan D.E., Furth E., Hunt S.J., Gade T.P.F. (2022). Transarterial Embolization Modulates the Immune Response within Target and Nontarget Hepatocellular Carcinomas in a Rat Model. Radiology.

[19] Cholongitas E., Papatheodoridis G.V., Vangeli M., Terreni N., Patch D., Burroughs A.K. (2005). Systematic Review: The Model for End-Stage Liver Disease--Should It Replace Child-Pugh’s Classification for Assessing Prognosis in Cirrhosis?. Aliment. Pharmacol. Ther..

[20] Hiraoka A., Michitaka K., Kumada T., Izumi N., Kadoya M., Kokudo N., Kubo S., Matsuyama Y., Nakashima O., Sakamoto M. (2017). Validation and Potential of Albumin-Bilirubin Grade and Prognostication in a Nationwide Survey of 46,681 Hepatocellular Carcinoma Patients in Japan: The Need for a More Detailed Evaluation of Hepatic Function. Liver Cancer.

[21] Freites-Martinez A., Santana N., Arias-Santiago S., Viera A. (2021). Using the Common Terminology Criteria for Adverse Events (Ctcae—Version 5.0) to Evaluate the Severity of Adverse Events of Anticancer Therapies. Actas Dermosifiliogr..

[22] Lencioni R., Llovet J.M. (2010). Modified Recist (Mrecist) Assessment for Hepatocellular Carcinoma. Semin. Liver Dis..

[23] Kudo M. (2024). A Changing Role of Transarterial Chemoembolization in the Era of Immune Checkpoint Inhibitor Plus Anti-Vegf/Tki Plus Transarterial Chemoembolization: From Total Embolization to Partial Embolization (Immune Boost Transarterial Chemoembolization). Liver Cancer.

[24] Shimose S., Kawaguchi T., Iwamoto H., Niizeki T., Shirono T., Tanaka M., Koga H., Torimura T. (2020). Indication of Suitable Transarterial Chemoembolization and Multikinase Inhibitors for Intermediate Stage Hepatocellular Carcinoma. Oncol. Lett..

[25] Zheng Y., Xiang Y., Shi H., Lin Z., Cheng S., Zhu J. (2024). Transarterial Chemoembolization Combined with Atezolizumab Plus Bevacizumab Versus Transarterial Chemoembolization Alone in Intermediate-Stage Hepatocellular Carcinoma: A Multicenter Retrospective Study. J. Hepatocell. Carcinoma.

[26] Shimose S., Iwamoto H., Niizeki T., Tanaka M., Shirono T., Moriyama E., Noda Y., Nakano M., Suga H., Kuromatsu R. (2023). Efficacy of Lenvatinib Combined with Transcatheter Intra-Arterial Therapies for Patients with Advanced-Stage of Hepatocellular Carcinoma: A Propensity Score Matching. Int. J. Mol. Sci..

[27] Uchino K., Tateishi R., Shiina S., Kanda M., Masuzaki R., Kondo Y., Goto T., Omata M., Yoshida H., Koike K. (2011). Hepatocellular Carcinoma with Extrahepatic Metastasis: Clinical Features and Prognostic Factors. Cancer.

[28] Iwamoto H., Shimose S., Niizeki T., Koga H., Torimura T. (2022). Clinical Significance of the Discrepancy between Radiological Findings and Biochemical Responses in Atezolizumab Plus Bevacizumab for Hepatocellular Carcinoma. Clin. Mol. Hepatol..

[29] Kudo M., Yamashita T., Finn R.S., Galle P., Ducreux M., Cheng A.-L., Tsuchiya K., Sakamoto N., Hige S., Take R. (2023). IMbrave150: Exploratory analyses for investigating associations between overall survival (OS) and depth of response (DpR) or duration of response (DoR) in patients (pts) with unresectable hepatocellular carcinoma (HCC). Ann. Oncol..

[30] Sergio A., Cristofori C., Cardin R., Pivetta G., Ragazzi R., Baldan A., Girardi L., Cillo U., Burra P., Giacomin A. (2008). Transcatheter Arterial Chemoembolization (Tace) in Hepatocellular Carcinoma (Hcc): The Role of Angiogenesis and Invasiveness. Am. J. Gastroenterol..

[31] Kudo M. (2024). Immune Checkpoint Inhibitors Plus Anti-Vegf/Tyrosine Kinase Inhibitors Combined with Tace (Triple Therapy) in Unresectable Hepatocellular Carcinoma. Liver Cancer.

[32] Hiraoka A., Kumada T., Michitaka K., Kudo M. (2019). Newly Proposed Albi Grade and Albi-T Score as Tools for Assessment of Hepatic Function and Prognosis in Hepatocellular Carcinoma Patients. Liver Cancer.

[33] Liang Y., Gan L., Zeng D., Lin L., Xiong Z., Liao F., Wang A. (2024). Clinical Efficacy of Lenvatinib, Trans-Arterial Chemoembolization, and Pd-1/L1 Inhibitors in Advanced Hepatocellular Carcinoma: A Systematic Review and Network Meta-Analysis. Clin. Transl. Oncol..

[34] Lencioni R., Kudo M., Erinjeri J., Qin S., Ren Z., Chan S., Sangro B. (2024). EMERALD-1: A phase 3, randomized, placebo-controlled study of transarterial chemoembolization combined with durvalumab with or without bevacizumab in participants with unresectable hepatocellular carcinoma eligible for embolization. J. Clin. Oncol..

[35] Llovet J.M., Finn R.S., Ren Z., Guo Y., Han G., Lin H., Kudo M. (2024). Transarterial chemoembolization (TACE) with or without lenvatinib (len) + pembrolizumab (pembro) for intermediate-stage hepatocellular carcinoma (HCC): Phase III LEAP-012 study. Ann. Oncol..

[36] Yamashita T., Inaba Y., Ikeda M., Sone M., Yamakado K., Nishiofuku H., Kudo M. (2023). IMPACT: Randomized, multicenter, phase III study evaluating the efficacy of immunotherapy (atezolizumab) plus anti-VEGF therapy (bevacizumab) in combination with transcatheter arterial chemoembolization for unresectable hepatocellular carcinoma (HCC). Ann. Oncol..

